# Effects of Ag^0^-modification and Fe^3+^-doping on the structural, optical and photocatalytic properties of TiO_2_

**DOI:** 10.1039/c9ra08655b

**Published:** 2019-12-04

**Authors:** Xiaodong Zhu, Hongyan Xu, Yin Yao, Hui Liu, Juan Wang, Yun Pu, Wei Feng, Shanhua Chen

**Affiliations:** College of Mechanical Engineering, Chengdu University Chengdu 610106 China fengwei233@126.com; College of Materials and Chemistry & Chemical Engineering, Chengdu University of Technology Chengdu 610059 China Chensh@cdut.edu.cn

## Abstract

Pure TiO_2_, Ag^0^-modified TiO_2_, Fe^3+^-doped TiO_2_, and Ag^0^-modified/Fe^3+^-doped TiO_2_ photocatalysts were synthesized *via* sol–gel technology. The crystal structure, element composition and surface morphology of the obtained photocatalysts were characterized *via* XRD, XPS, SEM and TEM, respectively. The results indicate that Ag–TiO_2_ samples show higher photocatalytic activity than pure TiO_2_. Unexpectedly, the photocatalytic activities of Fe–TiO_2_ and 1% Ag/1% Fe–TiO_2_ are lower than pure TiO_2_. To analyze the main factors affecting photocatalytic performance, the samples were further investigated by PL, DRS and BET. The results prove that the additions of Ag and Fe are advantageous for inhibiting the recombination of photoinduced pairs and improving the utilization of light. Fe–TiO_2_ and 1% Ag/1% Fe–TiO_2_ exhibit smaller specific surface areas than pure TiO_2_, which is the primary reason for their reduced photocatalytic performances.

## Abstract 2

The reasons for the photocatalytic activity of 1% Ag–TiO_2_ > pure TiO_2_ > 1% Ag/1% FeTiO_2_ > 1% Fe–TiO_2_ are investigated systematically.

## Graphical abstract

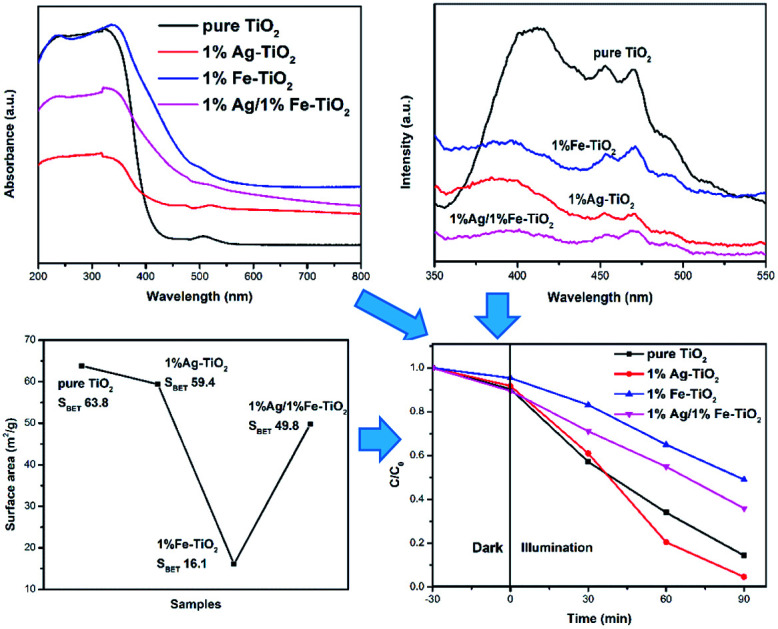

## Introduction

In the last few years, titanium dioxide (TiO_2_) has been widely used in the degradation of organic pollutants due to its strong photocatalytic ability, low cost, non-toxicity and chemical stability.^1–4^ However, two main drawbacks limit its practical application. Firstly, with a wide energy gap of 3.2 eV, TiO_2_ only absorbs ultraviolet light with a wavelength of less than 387 nm, therefore, the utilization rate of sunlight is limited.^5–9^ Secondly, the high recombination rate of photogenerated electrons and holes results in low quantum yield.^10–12^

Precious metal deposition is able to improve the utilization of sunlight and promote the separation of photogenerated pairs, which are in favor of photocatalytic activity.^13–16^ Zhou *et al.*^17^ prepared Au-deposited TiO_2_ films on indium–tin oxide glass by magnetron sputtering. Compared with pure TiO_2_ film, Au/TiO_2_ films show better photocatalytic activity because of their higher separation rate of photogenerated pairs and narrower band gaps. In the research of noble metal modifying, Ag-modified TiO_2_ has attracted much attention owing to its cheapness and effectiveness.^18^ Wang *et al.*^19^ prepared Ag/TiO_2_ nanotubes by electrospinning method and the photocatalytic performance is improved after Ag modification.

Besides, TiO_2_ modification by metal ion doping has also been extensively studied.^20–24^ Wu *et al.*^25^ synthesized Cu-doped TiO_2_ by hydrothermal synthesis and air heat treatment. 0.5 mol%-Cu/TiO_2_ shows better photocatalytic activity than pure TiO_2_ because it enlarges the visible light absorbance due to the presence of Cu 3d orbitals. However, the photocatalytic performance declines when the concentration of Cu is more than 1 mol%. They believe that the high content of Cu forms new recombination centers, reducing the separation of photoinduced pairs. Moradi *et al.*^26^ reported that Fe-doped TiO_2_ shows higher visible light photocatalytic activity than pure TiO_2_ owing to its red-shift and lower recombination rate. However, there are also studies have shown that the photocatalytic activity of TiO_2_ is reduced after metal ion doping.^23,27^ For instance, Kundu *et al.*^27^ synthesized Fe-doped and pure TiO_2_ nanoparticles and it is found that pure TiO_2_ shows the highest degradation rate of MB under sunlight. Cu-doped TiO_2_ films were prepared by sol–gel dip-coating method, and their photodegradation rates are lower than that of pure TiO_2_ film, which has been reported by Bensouici *et al.*^23^

Compared with single element modification, multi-elements may produce a synergistic effect and further improve the photocatalytic activity of TiO_2_.^1,4,7,22,28^ Zhang *et al.*^28^ synthesized Ln^3+^/Ag^0^–TiO_2_, Ln^3+^–TiO_2_, Ag^0^–TiO_2_ and pure TiO_2,_ and photocatalytic tests show that Ln^3+^/Ag^0^–TiO_2_ exhibits the best photocatalytic activity. Ln^3+^/Ag^0^–TiO_2_ presents the lowest PL intensity because the synergistic effect of Ln^3+^ doping and Ag^0^ deposition provides more trap centers, which promotes the transfer of photoinduced electrons, suppressing the charge recombination effectively. Meanwhile, some studies have shown that single element displays better modification effect than multi-elements.^24,29^ Khan *et al.*‘s research^29^ indicates that Ga-doped TiO_2_ exhibits better photocatalytic performance than N/Ga co-doped TiO_2_. Malengreaux *et al.*^24^ reported that Eu/Fe co-doped TiO_2_ shows lower photocatalytic efficiency than Fe-doped TiO_2_ and pure TiO_2_.

Therefore, to explore the effects of single element modification and two elements co-modification on the photocatalytic performance of TiO_2_, the pure, Ag-modified, Fe-doped and Ag/Fe-modified TiO_2_ were prepared and their photocatalytic activities were investigated. The effects of Ag and Fe addition on the structure, morphology, optical and photocatalytic properties of TiO_2_ were analyzed systematically.

## Experimental

### Preparation of materials

**Fig. 1:**
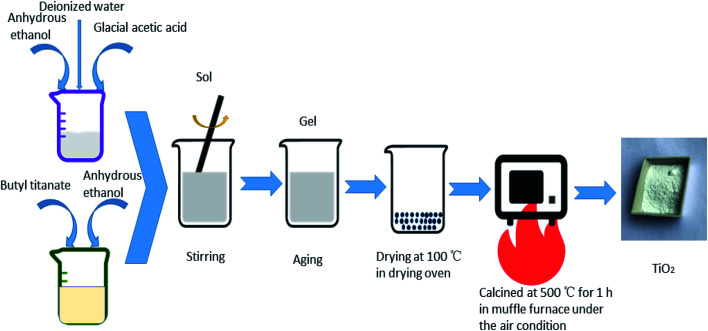
The preparation process of TiO_2_.

All the TiO_2_ photocatalysts were prepared by sol–gel method. The specific process of preparation pure TiO_2_ was as follows: solution A was prepared by adding butyl titanate in a volume ratio of 1 : 2 to anhydrous ethanol in a beaker. Solution B was prepared by adding anhydrous ethanol, glacial acetic acid and deionized water in a volume ratio of 4 : 5 : 5. Solution B was added to solution A dropwise. The resulting mixture was stirred until a sol formed. A gel formed after aging. The obtained gel was dried at 100 °C and then heat treated at 500 °C for 1 h to obtain pure TiO_2_. For the preparation of Ag-modified TiO_2_ and Fe-doped TiO_2_, certain amounts of AgNO_3_ or Fe(NO_3_)_3_·9H_2_O were added into solution B. The atomic percentages of Ag/Ti or Fe/Ti were 0.5%, 1%, 2% and 4%, respectively. Keep other steps unchanged to obtain different concentrations of Ag modified TiO_2_ and Fe doped TiO_2_ which are recorded as 0.5% Ag–TiO_2_, 1% Ag–TiO_2_, 2% Ag–TiO_2_, 4% Ag–TiO_2_, 0.5% Fe–TiO_2_, 1% Fe–TiO_2_, 2% Fe–TiO_2_ and 4% Fe–TiO_2_. AgNO_3_ and Fe(NO_3_)_3_·9H_2_O were simultaneously added into solution B to prepare Ag/Fe co-modified TiO_2_. Both the molar ratios of Ag/Ti and Fe/Ti are 1%, and the Ag/Fe co-modified TiO_2_ is labeled as 1% Ag/1% Fe–TiO_2_. The preparation process is shown in Fig. 1.

### Characterization

The phase structure of photocatalysts was determined using an X-ray diffractometer (XRD). Element composition and valence state were analyzed by an X-ray photoelectron spectroscopy (XPS). Surface morphologies were observed by a field emission scanning electron microscopy (FESEM) and a transmission electron microscopy (TEM). Photoluminescence (PL) spectra were recorded through a luminescence spectrometer. UV-vis diffuse reflectance spectra (DRS) were recorded using a spectrophotometer. Specific surface areas were determined by the Brunauer Emmett Teller (BET) method.

### Photocatalytic test

Add 100 mL rhodamine B (RhB) solution (10 mg L^−1^) and 0 1 g of TiO_2_ photocatalyst to the beaker. The mixture was ultrasonically dispersed for 10 minutes, and then stirred to establish the adsorption–desorption equilibrium in dark for 30 min. A 250 W xenon lamp was employed as a light source and a small amount of suspension was taken every 30 minutes. After centrifugation, the supernatant was extracted and the absorbance of RhB was measured with a spectrophotometer at the wavelength of 553 nm. The decolorization rate *D* (%) was calculated as follows:*D* = (1−*A*_*t*_/*A*_0_)

In the formula, *A*_0_ is the initial absorbance, and *A*_*t*_ is the absorbance at time *t*.

## Results and discussion

### XRD analysis

**Fig. 2:**
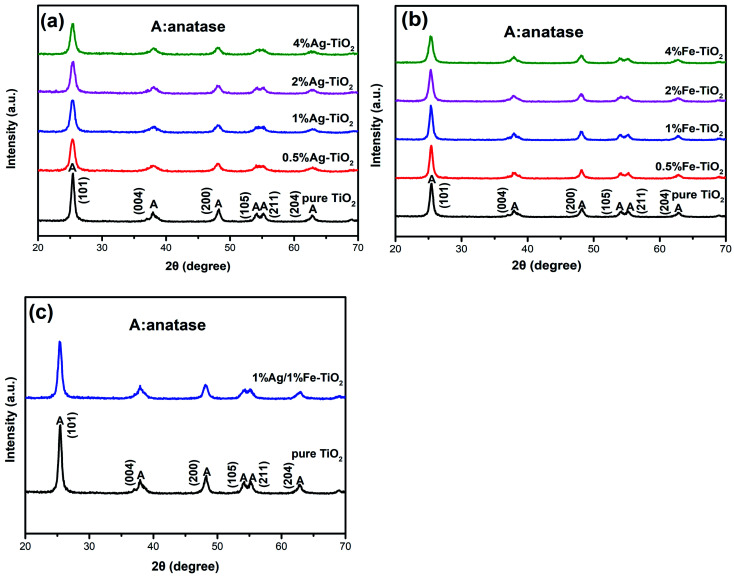
XRD patterns of (a) Ag–TiO_2_, (b) Fe–TiO_2_ and (c) 1% Ag/1% Fe–TiO_2_.

Fig. 2 shows the XRD patterns of pure TiO_2_, Ag–TiO_2_, Fe–TiO_2_ and 1% Ag/1% Fe–TiO_2_. In Fig. 2(a), the peaks of pure TiO_2_ at 25.3°, 37.9°, 48.1°, 53.9°, 55.1°and 62.8° correspond to (101), (004), (200), (105), (211) and (204) crystal planes of anatase structure, respectively. It is observed that Ag–TiO_2_, Fe–TiO_2_ and 1% Ag/1% Fe–TiO_2_ also form anatase structure, suggesting that the crystal structure of TiO_2_ is not significantly affected by Ag or Fe adding. However, the widths of diffraction peaks in Ag–TiO_2_, Fe–TiO_2_ and 1% Ag/1% Fe–TiO_2_ are wider and the intensities are lower than pure TiO_2_, indicating that the crystallinity of TiO_2_ is reduced and the crystal grain is refined.^26,30^ The grain sizes of TiO_2_ are calculated by Scherrer formula^21^ and the results are shown in Table 1. Yu *et al.*^31^ believe that the addition of dopants hinders the contact between TiO_2_ particles and inhibits the growth of grains during the heat treatment process, resulting in the decrease of grain size. The absence of diffraction peaks associated with Fe may be attributed to the fact that Fe^3+^ ions can replace Ti^4+^ ions into TiO_2_ lattices due to their close ionic radius.^26,32,33^ On the other hand, since the radius of Ag atom is much larger than that of Ti^4+^, it is difficult to enter TiO_2_ lattices. Ag particles probably disperse on TiO_2_ surface in the form of metallic Ag.^34,35^

**Table 1:** The crystallite sizes of samples

Samples	Crystallite size (nm)	Samples	Crystallite size (nm)
Pure TiO_2_	16.0	0.5% Fe–TiO_2_	15.1
0.5% Ag–TiO_2_	11.2	1% Fe–TiO_2_	15.9
1% Ag–TiO_2_	11.6	2% Fe–TiO_2_	13.7
2% Ag–TiO_2_	11.9	4% Fe–TiO_2_	12.3
4% Ag–TiO_2_	12.0	1% Ag/1% Fe–TiO_2_	13.3

### XPS analysis

**Fig. 3:**
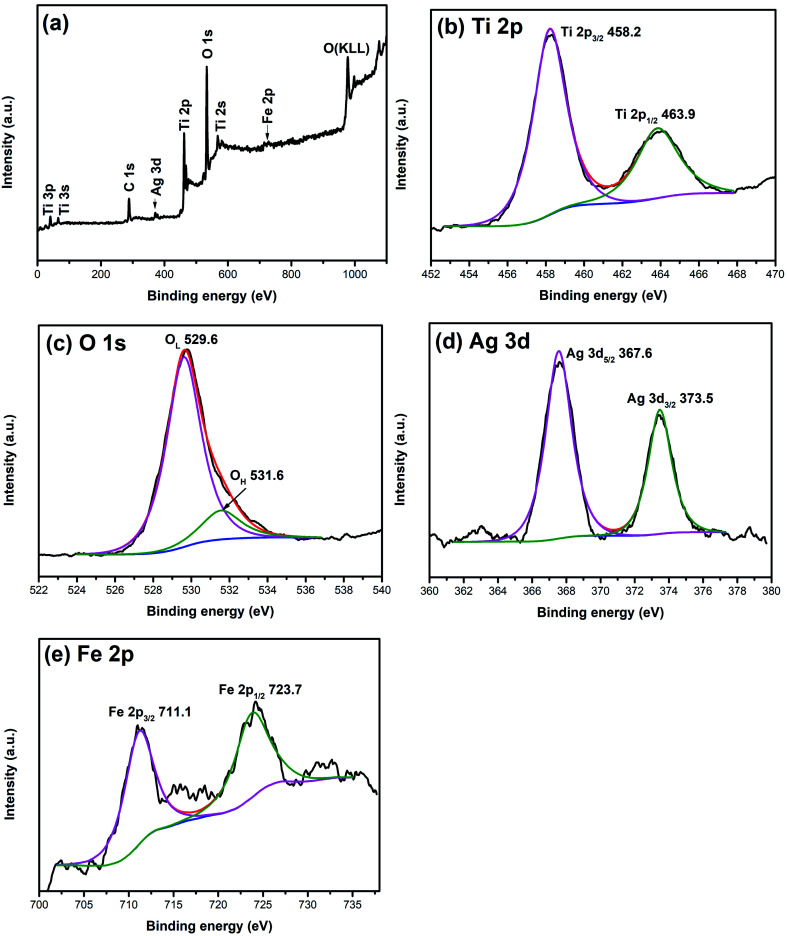
XPS spectra of 1% Ag/1% Fe–TiO_2_: (a) total spectrum, (b) Ti 2p, (c) O 1s, (d) Ag 3d and (e) Fe 2p.

XPS is widely used to investigate the chemical valence of elements. To verify the valence states of Ag and Fe elements, XPS analysis of 1% Ag/1% Fe–TiO_2_ has been carried out and the results are shown in Fig. 3. The C, Ti, O, Ag and Fe signal peaks appear in the full spectrum (Fig. 3(a)), meaning that there are C, Ti, O, Ag and Fe elements in the sample. The appearance of C 1s peak may be derived from C remaining in the organic calcination process. In Fig. 3(b), two peaks at 458.2 eV and 463.9 eV corresponding to Ti 2p_3/2_ and Ti 2p_1/2_ and the splitting energy between Ti 2p_3/2_ and Ti 2p_1/2_ (5.7 eV) indicate that the state of Ti ions is Ti^4+^.^36–38^Fig. 3(c) presents two peaks at 529.6 eV and 531.6 eV, ascribing to Ti–O bonds in TiO_2_ lattices (O_L_) and surface hydroxyl groups (O_H_), respectively.^39,40^ In Fig. 3(d), the peaks at 367.6 eV and 373.5 eV can be assigned to Ag 3d_5/2_ and Ag 3d_3/2_, respectively, which suggests that the Ag element exists as Ag^0^ in the sample.^34,41,42^ Two peaks at 711.1 eV and 723.7 eV in Fig. 3(e) attribute to Fe 2p_3/2_ and Fe 2p_1/2_, which indicates Fe element exists as Fe^3+^.^27,32,43,44^

### SEM and TEM images

**Fig. 4:**
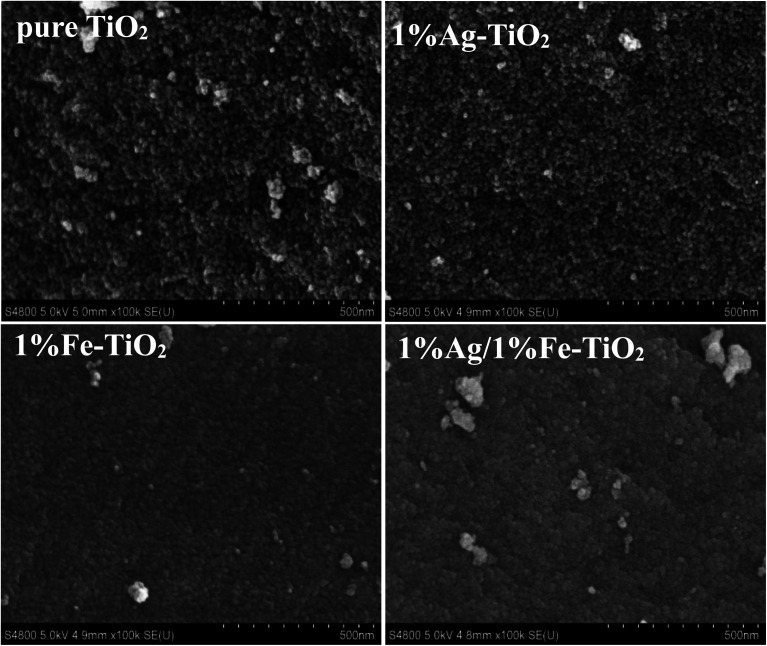
SEM images of pure TiO_2_, 1% Ag–TiO_2_, 1% Fe–TiO_2_ and 1% Ag/1% Fe–TiO_2_.

Fig. 4 presents the SEM images of pure TiO_2_, 1% Ag–TiO_2_, 1% Fe–TiO_2_, and 1% Ag/1% Fe–TiO_2_. It is observed that the particles are nearly spherical. Among them, pure TiO_2_ and 1% Ag–TiO_2_ particles are fine and disperse relatively uniform, however, the particles in 1% Fe–TiO_2_ and 1% Ag/1% Fe–TiO_2_ present obvious agglomeration.

**Fig. 5:**
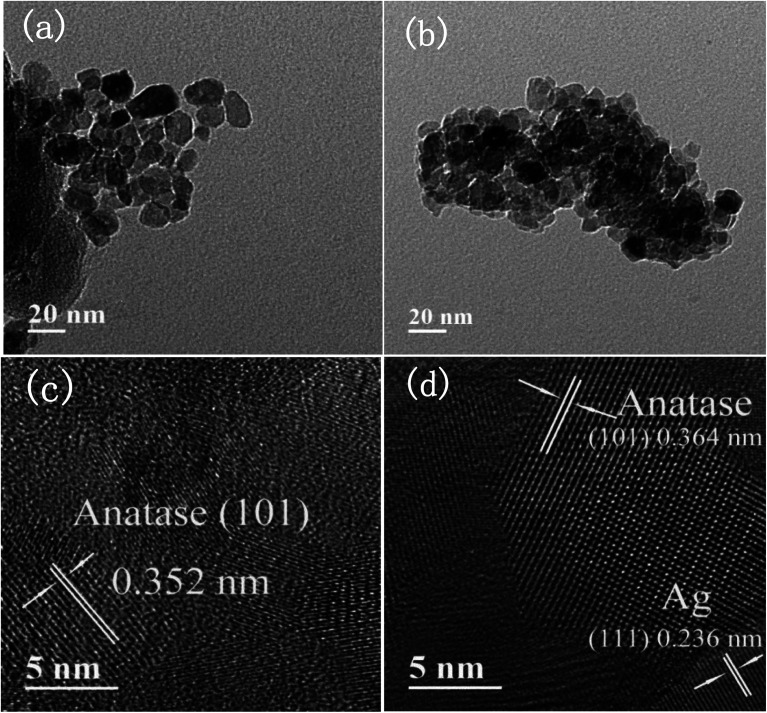
TEM images of (a) pure TiO_2_, (b) 1% Ag/1% Fe–TiO_2_, HRTEM images of (c) pure TiO_2_, and (d) 1% Ag/1% Fe–TiO_2_.

Fig. 5(a) and (b) are TEM images of pure TiO_2_ and 1% Ag/1% Fe–TiO_2_, from which we can see that the particle size in pure TiO_2_ is about 15–20 nm, while it is arranged from 10 to 15 nm in 1% Ag/1% Fe–TiO_2_. Moreover, pure TiO_2_ shows better particle dispersion than 1% Ag/1% Fe–TiO_2_, which is consistent with SEM images. The HRTEM images of pure TiO_2_ and 1% Ag/1% Fe–TiO_2_ are presented in Fig. 5(c) and (d), respectively. Both the labeled interplanar spacing values in pure TiO_2_ (0.352 nm) and 1% Ag/1% Fe–TiO_2_ (0.364 nm) correspond to the anatase (101) crystal plane.^45^ Since the radius of Fe^3+^ ions is larger than of Ti^4+^ ions, the substitution of Fe^3+^ for Ti^4+^ into crystal lattices causes lattice expansion, consequently, the anatase (101) plane spacing of 1% Ag/1% Fe–TiO_2_ is slightly larger than that of pure TiO_2_.^25^ In Fig. 5(d), the marked interplanar spacing (0.236 nm) can be attributed to the (111) crystal plane of metallic Ag^0^,^34,39^ which is in accord with XPS results.

### Photocatalytic performance

#### Photodegradation results

**Fig. 6:**
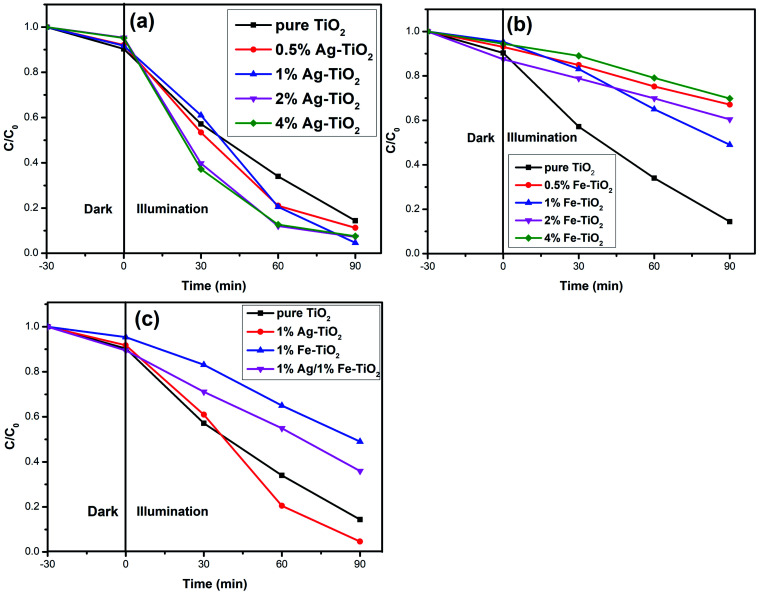
Decolorization rate curves of RhB for (a) Ag–TiO_2_, (b) Fe–TiO_2_, and (c) 1% Ag/1% Fe–TiO_2_.

**Fig. 7:**
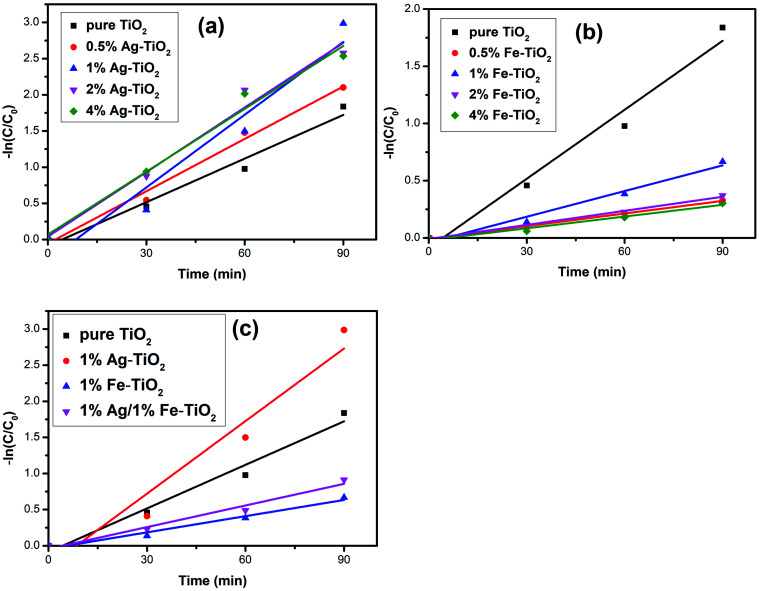
Kinetics linear simulation curves of (a) Ag–TiO_2_, (b) Fe–TiO_2_, and (c) 1% Ag/1% Fe–TiO_2_.

The photocatalytic activity of photocatalysts was evaluated *via* the decolorization rate of RhB and the results are presented in Fig. 6. The RhB decolorization rate of pure TiO_2_ is 85.7% after 90 min of reaction. Ag–TiO_2_ samples show higher photocatalytic activity than pure TiO_2_. The decolorization rates of 0.5% Ag–TiO_2_, 1% Ag–TiO_2_, 2% Ag–TiO_2_ and 4% Ag–TiO_2_ are 88.8%, 95.4%, 92.8% and 92.5%, respectively. 1% Ag–TiO_2_ exhibits the best photocatalytic activity and the decolorization rate of RhB decreases when Ag content exceeds 1%. Unexpectedly, the photocatalytic activity of TiO_2_ decreases after Fe doping. The decolorization rates of 0.5% Fe–TiO_2_, 1% Fe–TiO_2_, 2% Fe–TiO_2_ and 4% Fe–TiO_2_ are 33.3%, 51.3%, 39.9% and 30.6%, respectively. It is clear that the addition of Ag improves the photocatalytic performance, while Fe is the opposite. The decolorization rate of 1% Ag/1% Fe–TiO_2_ is 64.3%, suggesting that its photocatalytic activity is higher than Fe–TiO_2_ but lower than pure TiO_2_. The decolorization of RhB by TiO_2_ can be considered as a first-order reaction,^17,19,46^ and the calculation formula of the reaction rate constant *k* is as follows:ln(*C*_*t*_/*C*_0_) = −*kt*where *t* is the reaction time, *C*_0_ and *C*_*t*_ are the initial concentration and the concentration at time *t*. The first-order reaction kinetics fit curves are shown in Fig. 7. It is calculated that the reaction rate constant of pure TiO_2_ is 0.020 min^−1^. Ag–TiO_2_ samples show higher reaction rates than pure TiO_2_, and the reaction rate constants of 0.5% Ag–TiO_2_, 1% Ag–TiO_2_, 2% Ag–TiO_2_ and 4% Ag–TiO_2_ are 0.024 min^−1^, 0.034 min^−1^, 0.030 min^−1^ and 0.029 min^−1^, respectively. The reaction rate constants of 0.5% Fe–TiO_2_, 1% Fe–TiO_2_, 2% Fe–TiO_2_ and 4% Fe–TiO_2_ are 0.0036 min^−1^, 0.0075 min^−1^, 0.0041 min^−1^ and 0.0034 min^−1^, respectively, implying that the reaction rates of Fe–TiO_2_ are much lower than pure TiO_2_. The reaction rate constant of 1% Ag/1% Fe–TiO_2_ is 0.0099 min^−1^, which is higher than Fe–TiO_2_ but lower than pure TiO_2_.

### PL spectra

**Fig. 8:**
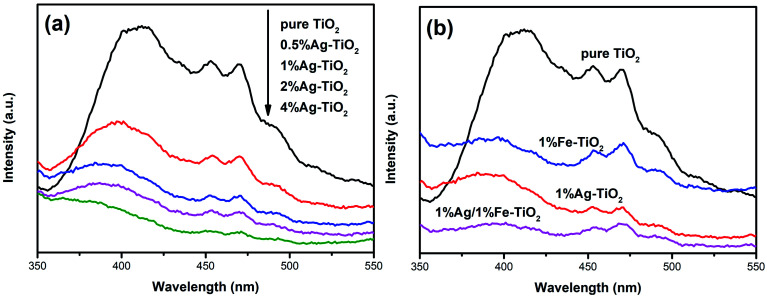
PL spectra of (a) Ag–TiO_2_, (b) pure TiO_2_, 1% Ag–TiO_2_, 1% Fe–TiO_2_ and 1% Ag/1% Fe–TiO_2_.

In order to analyze the influence of addition with Ag and Fe on the photogenerated electron–hole recombination of TiO_2_, the PL tests have been implemented and the results are shown in Fig. 8. The PL peaks originate from the recombination of photoinduced electrons and holes. Therefore, a lower PL peak intensity represents a lower recombination rate.^27,38^ The PL peak intensity of Ag–TiO_2_ is lower than that of pure TiO_2_, which suggests that the recombination of photoinduced pairs is inhibited by Ag modification. When exposed to light source, electrons in the valence band are excited to the conduction band, forming photogenerated electrons, leaving photogenerated holes on valence band. Photoinduced electrons can be transferred to Ag^0^ particles which are deposited on TiO_2_ surface, reducing the recombination.^6,18,19,34^ Several researches have reported that there is an optimum concentration of Ag, above which new recombination centers will be formed thus increases recombination rate.^18,34^ However, PL peak intensity decreases with the increasing Ag concentration in the present work, indicating that new recombination centers have not been formed when the Ag concentration reaches 4%. The PL peak intensity decreases with the increase of the amount of noble metal element, which also has been reported in previous work.^47^ Photocatalytic experiments show that high Ag concentration is not conducive to photocatalytic activity and 1% Ag–TiO_2_ exhibits the highest decolorization rate. The decrease in photocatalytic performance of 2% Ag–TiO_2_ and 4% Ag–TiO_2_ should be attributed to the fact that Ag^0^ particles are deposited on the surface of TiO_2_ particles, and as the Ag concentration increases, excessive Ag particles will cover TiO_2_ surface, reducing the utilization of light and reactive sites.^13,34^

The photocatalytic activities of Fe–TiO_2_ and 1% Ag/1% Fe–TiO_2_ are lower than pure TiO_2_. Similar results have also been reported that the addition of metal ions reduces the photocatalytic performance of TiO_2_.^23^ There is a viewpoint that doping metal ions leads to a decrease in crystallinity and an increase in lattice defects. The formed lattice defects act as recombination centers, promoting the recombination of photoinduced pairs and suppressing the photocatalytic activity.^23,48,49^ In contrast, from Fig. 8(b), it is clear that the PL intensities of 1% Fe–TiO_2_ and 1% Ag/1% Fe–TiO_2_ are lower than that of pure TiO_2_, proving that the addition of Fe is beneficial to decreasing the recombination. Fe^3+^ ions entering the crystal lattices to replace Ti^4+^ ions will cause lattice distortion and defects, capturing photogenerated electrons and reducing the recombination of photogenerated charges.^7,33^ Moreover, 1% Ag/1% Fe–TiO_2_ shows the lowest PL intensity, suggesting that there is a synergistic effect in suppressing photoinduced pairs recombination owing to adding Ag and Fe simultaneously. Therefore, we can conclude that the decrease in photocatalytic performance of Fe–TiO_2_ and 1% Ag/1% Fe–TiO_2_ should not be attributed to the promotion of recombination with Fe adding.

### DRS analysis

**Fig. 9:**
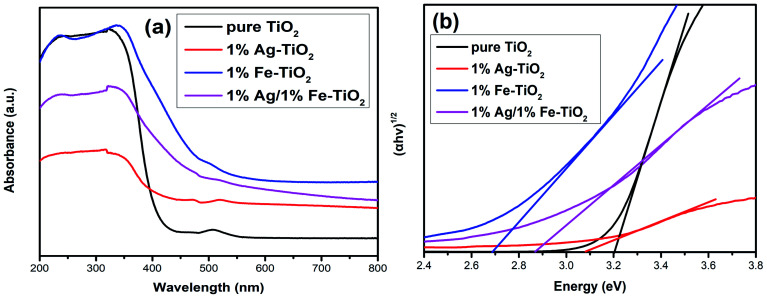
(a) UV-visible absorption spectra and (b) plots of (*αhν*)^1/2^*versus* energy (*hν*) of pure TiO_2_, 1% Ag–TiO_2_, 1% Fe–TiO_2_ and 1% Ag/1% Fe–TiO_2_.

The energy gap (*E*_g_) of photocatalyst affects the absorption of light source, which is an important factor for photocatalytic performance. The influence of doping on the *E*_g_ of TiO_2_ is controversial, and both redshift^6,7,9,26,27,34^ and blueshift^24^ have been reported. Fig. 9 depicts the UV-visible absorption spectra of pure TiO_2_ and 1% Ag–TiO_2_, 1% Fe–TiO_2_ and 1% Ag/1% Fe–TiO_2_. It is observed that the addition of Ag and Fe causes a redshift in the absorption edge of TiO_2_. The *E*_g_ was calculated based on the Kubelka–Munk equation and Tauc's plots.^3,32,35,50^ The *E*_g_ of pure TiO_2_, 1% Ag–TiO_2_, 1% Fe–TiO_2_ and 1% Ag/1% Fe–TiO_2_ are estimated to be 3.20 eV, 3.09 eV, 2.69 eV and 2.87 eV, respectively. The results show that the addition of Ag and Fe is beneficial to increasing the absorption of visible light. It can be concluded from XPS and TEM results that Ag element exists in the form of Ag^0^. Due to the surface plasmon resonance (SPR) effect of Ag particles deposited on TiO_2_ surface, the absorption of visible light can be enhanced, reducing the *E*_g_.^15,16,34,36,37^ On the other hand, the substitutions of Ti^4+^ ions with Fe^3+^ ions form impurity energy levels between the conduction band and valence band in the forbidden band, thereby decreasing the *E*_g_.^5,7,10,21,33,43,51^ Therefore, the change of *E*_g_ is not the main reason for the decreased photocatalytic performance of Fe–TiO_2_ and 1% Ag/1% Fe–TiO_2_.

### BET analysis

As is well known, photocatalyst with a larger specific surface area will provide more active reaction sites and increase light absorption, which is advantageous for photocatalytic performance.^19,22,27,33^ It is observed from SEM images that the 1% Fe–TiO_2_ particles have a larger agglomeration than pure TiO_2_, which may lead to the decrease of specific surface area. To verify this assumption, we have performed BET specific surface area tests and the results are shown in Table 2. The specific surface area of 1% Fe–TiO_2_ is 16.1 m^2^ g^−1^, which is much lower than that of pure TiO_2_ (63.8 m^2^ g^−1^). Some researchers believe that reduction in grain size will lead to an increase in specific surface area.^48,51^ In our work, the grain size of TiO_2_ is reduced by Ag or Fe modification, which is proved by XRD results, however, 1% Fe–TiO_2_ and 1% Ag/1% Fe–TiO_2_ possess smaller specific surface areas than pure TiO_2_. Wang *et al.*^52^ and Adyani *et al.*^22^ are convinced that the specific surface area of TiO_2_ is largely related to agglomeration degree. SEM and TEM images confirm an increase in particle agglomeration after Fe addition, which is the major reason for the decrease in specific surface area. It is the significant reduce in specific surface areas of Fe–TiO_2_ and 1% Ag/1% Fe–TiO_2_ that causes the decrease in their photocatalytic performance.

**Table 2:** Specific surface areas (*S*_BET_) of pure TiO_2_, 1% Ag–TiO_2_, 1% Fe–TiO_2_ and 1% Ag/1% Fe–TiO_2_

Samples	*S*_BET_ (m^2^ g^−1^)
Pure TiO_2_	63.8
1% Ag–TiO_2_	59.4
1% Fe–TiO_2_	16.1
1% Ag/1% Fe–TiO_2_	49.8

From another perspective, as the surface areas of samples are quite different, the intrinsic photocatalytic activity (normalized by BET surface area) can be used as a reference. The intrinsic photocatalytic activity (*I*_PA_) is calculated as follows:^53^*I*_PA_ (mg min^−1^ m^−2^) = *m*_RhB_/*tS*_BET_*m*_s_where *m*_RhB_ is the weight of RhB which has been degraded, *t* is the reaction time (90 min), *S*_BET_ is the surface area, and *m*_s_ is the weight of sample used in each test (0.1 g).

The *I*_PA_ of pure TiO_2_, 1% Ag–TiO_2_, 1% Fe–TiO_2_ and 1% Ag/1% Fe–TiO_2_ are 0.00149 mg min^−1^ m^−2^, 0.00178 mg min^−1^ m^−2^, 0.00354 mg min^−1^ m^−2^ and 0.00143 mg min^−1^ m^−2^. The results show that 1% Fe–TiO_2_ has relatively high intrinsic photocatalytic activity.

## Conclusions

In summary, pure TiO_2_, Ag–TiO_2_, Fe–TiO_2_ and 1% Ag/1% Fe–TiO_2_ were prepared by sol–gel method. The results of the decolorization rate of RhB indicate that the photocatalytic activities of Ag–TiO_2_ are higher than pure TiO_2_, while the photocatalytic activities of Fe–TiO_2_ and 1% Ag/1% Fe–TiO_2_ are lower than pure TiO_2_. The increased photocatalytic activity of Ag–TiO_2_ can be attributed to the reduction of photoinduced pairs recombination rate and energy gap. The specific surface areas of Fe–TiO_2_ and 1% Ag/1% Fe–TiO_2_ are much lower than pure TiO_2_, leading to the decreases in their photocatalytic properties.

## Conflicts of interest

There are no conflicts to declare.

## Supplementary Material

## References

[cit1] Singaram B., Varadharajan K., Jeyaram J., Rajendran R., Jayavel V. (2017). J. Photochem. Photobiol., A.

[cit2] Koli V., Kim J. (2019). Mater. Sci. Semicond. Process..

[cit3] Pérez J. A. B., Courel M., Pal M., Delgado F. P., Mathews N. R. (2017). Ceram. Int..

[cit4] Gaikwad P. N., Hankare P. P., Wandre T. M., Garadkar K. M., Sasikala R. (2016). Mater. Sci. Eng., B.

[cit5] Loan T. T., Bang N. A., Huong V. H., Long N. N. (2017). Opt. Mater..

[cit6] Wei N., Cui H. Z., Song Q., Zhang L. Q., Song X. J., Wang K., Zhang Y. F., Li J., Wen J., Tian J. (2016). Appl. Catal., B.

[cit7] Benjwal P., De B., Kar K. K. (2018). Appl. Surf. Sci..

[cit8] Zhang Y. C., Afzal N., Pan L., Zhang X. W., Zou J. J. (2019). Adv. Sci..

[cit9] Ambati R., Gogate P. R. (2018). Ultrason. Sonochem..

[cit10] Zhang Y. Y., Gu D., Zhu L. Y., Wang B. H. (2017). Appl. Surf. Sci..

[cit11] Jiang H. B., Xing J., Chen Z. P., Tian F., Cuan Q., Gong X. Q., Yang H. G. (2014). Catal. Today.

[cit12] Li J. J., Deng X. Y., Guo R. N., Li B., Cheng Q. F., Cheng X. W. (2018). J. Taiwan Inst. Chem. Eng..

[cit13] Yang Z. L., Lu J., Ye W. C., Yu C. S., Chang Y. L. (2017). Appl. Surf. Sci..

[cit14] Lin W., Zheng H., Zhang P. Y., Xu T. Z. (2016). Appl. Catal., A.

[cit15] Naya S., Tada H. (2018). J. Catal..

[cit16] Sadrieyeh S., Malekfar R. (2018). J. Non-Cryst. Solids.

[cit17] Zhou D. Y., Liu Y. M., Zhang W. G., Liang W., Yang F. Q. (2017). Thin Solid Films.

[cit18] Ali T., Ahmed A., Alam U., Uddin I., Tripathi P., Muneer M. (2018). Mater. Chem. Phys..

[cit19] Wang T., Wei J. X., Shi H. M., Zhou M., Zhang Y., Chen Q., Zhang Z. M. (2017). Phys. E.

[cit20] Salazar-Villanueva M., Cruz-López A., Zaldívar-Cadena A. A., Tovar-Corona A., Guevara-Romero M. L., Vazquez-Cuchillo O. (2017). Mater. Sci. Semicond. Process..

[cit21] Arunachalam A., Dhanapandian S., Manoharan C. (2016). Phys. E.

[cit22] Adyani S. M., Ghorbani M. (2018). J. Rare Earths.

[cit23] Bensouici F., Bououdina M., Dakhel A. A., Tala-Ighil R., Tounane M., Iratni A., Souier T., Liu S., Cai W. (2017). Appl. Surf. Sci..

[cit24] Malengreaux C. M., Pirard S. L., Leonard G., Mahy J. G., Herlitschke M., Klobes B., Hermann R., Heinrichs B., Bartlett J. R. (2017). J. Alloys Compd..

[cit25] Wu M. C., Wu P. Y., Lin T. H., Lin T. F. (2017). Appl. Surf. Sci..

[cit26] Moradi H., Eshaghi A., Hosseini S. R., Ghani K. (2016). Ultrason. Sonochem..

[cit27] Kundu A., Mondal A. (2019). J. Mater. Sci.: Mater. Electron..

[cit28] Zhang P. F., Li X. W., Wu X. K., Zhao T. X., Wen L. S. (2016). J. Alloys Compd..

[cit29] Khan M., Yi Z., Gul S. R., Fawad U., Muhammad W. (2017). J. Phys. Chem. Solids.

[cit30] Chen Y., Liu K. (2017). J. Hazard. Mater..

[cit31] Yu J. G., Yu J. C., Zhao X. J. (2002). J. Sol-Gel Sci. Technol..

[cit32] Sood S., Umar A., Mehta S. K., Kansal S. K. (2015). J. Colloid Interface Sci..

[cit33] Li J. P., Ren D. J., Wu Z. X., Xu J., Bao Y. J., He S., Chen Y. H. (2018). J. Colloid Interface Sci..

[cit34] Lin X. X., Rong F., Fu D. G., Yuan C. W. (2012). Powder Technol..

[cit35] Naraginti S., Thejaswini T. V. L., Prabhakaran D., Sivakumar A., Satyanarayana V. S. V., Arun Prasad A. S. (2015). Spectrochim. Acta, Part A.

[cit36] Gao D. d., Liu W. J., Xu Y., Wang P., Fan J. J., Yu H. G. (2020). Appl. Catal., B.

[cit37] Fan X., Fan J., Hu X. Y., Liu E. Z., Kang L. M., Tang C. N., Ma Y. N., Wu H. T., Li Y. Y. (2014). Ceram. Int..

[cit38] Chen Y., Wu Q., Zhou C., Jin Q. T. (2017). Powder Technol..

[cit39] Zhang Y., Wang T., Zhou M., Wang Y., Zhang Z. M. (2017). Ceram. Int..

[cit40] Demirci S., Dikici T., Yurddaskal M., Gultekin S., Toparli M., Celik E. (2016). Appl. Surf. Sci..

[cit41] Yao Y. C., Dai X. R., Hu X. Y., Huang S. Z., Jin Z. (2016). Appl. Surf. Sci..

[cit42] Gao Y. P., Fang P. F., Chen F. T., Liu Y., Liu Z., Wang D. H., Dai Y. Q. (2013). Appl. Surf. Sci..

[cit43] Shen G. Q., Pan L., Lü Z., Wang C. Q., Aleem F., Zhang X. W., Zou J. J. (2018). Chin. J. Catal..

[cit44] Pan L., Zou J. J., Zhang X. W., Wang L. (2010). Ind. Eng. Chem. Res..

[cit45] Gao D. D., Wu X. H., Wang P., Xu Y., Yu H. G., Yu J. G. (2019). ACS Sustainable Chem. Eng..

[cit46] Zhang D., Wang B. H., Wang J. Q., Wang H. M., Zhang S. X., Gu D. (2019). RSC Adv..

[cit47] Yang X. J., Wu X. L., Li J., Liu Y. (2019). RSC Adv..

[cit48] Dao D. V., Bremt M. V. D., Koeller Z., Le T. K. (2016). Powder Technol..

[cit49] Le T. H., Bui A. T., Le T. K. (2014). Powder Technol..

[cit50] Tauc J., Grigorovici R., Vancu A. (1966). Phys. Status Solidi.

[cit51] Kalantari K., Kalbasi M., Sohrabi M., Royaee S. J. (2017). Ceram. Int..

[cit52] Wang Y. Z., Wu Y. S., Yang H., Xue X. X., Liu Z. H. (2016). Vacuum.

[cit53] Guo R. T., Chen Q. L., Ding H. L., Wang Q. S., Pan W. G., Yang N. Z., Lu C. Z. (2015). Catal. Commun..

